# Oral Administration of *Lactobacillus Casei* Expressing Flagellin A Protein Confers Effective Protection against *Aeromonas Veronii* in Common Carp, *Cyprinus Carpio*

**DOI:** 10.3390/ijms21010033

**Published:** 2019-12-19

**Authors:** Jia-Xin Tian, Yuan-Huan Kang, Guo-Sheng Chu, Hong-Jian Liu, Yi-Di Kong, Lin-Hui Zhao, Yu-Xin Kong, Xiao-Feng Shan, Gui-Qin Wang

**Affiliations:** 1 College of Animal Science and Technology, Jilin Agriculture University, Changchun 130118, China; 17767733220@163.com (J.-X.T.); kangyuanhuan@jlau.edu.cn (Y.-H.K.); kydjlau@sina.com (Y.-D.K.); ZLHJLAU@163.com (L.-H.Z.); bbxykyx@163.com (Y.-X.K.); 2 Ministry of Education Laboratory of Animal Production and Quality Security, Jilin Agricultural University Changchun, Jilin 130118, China; 3 Aquatic Production Technology Promotion Station of Jilin Province, Changchun 130012, China; cgsjlau@sina.com (G.-S.C.); lhjjlau@sina.com (H.-J.L.)

**Keywords:** oral administration, vaccine, *Aeromonas veronii*, *Lactobacillus casei*, immune response

## Abstract

*Aeromonas veronii* is a pathogen capable of infecting humans, livestock and aquatic animals, resulting in serious economic losses. In this study, two recombinant *Lactobacillus casei* expressing flagellin A (FlaA) of *A. veronii*, Lc-pPG-1-FlaA (surface-displayed) and Lc-pPG-2-FlaA (secretory) were constructed. The immune responses in fish administered with recombinant *L. casei* were evaluated. The two recombinant *L. casei* were orally administered to common carp, which stimulated high serum IgM and induced higher ACP, AKP, SOD and LYZ activity. Using qRT-PCR, the expression of IL-10, IL-8, IL-1β, TNF-α and IFN-γ in the tissue of fish immunized with recombinant *L. casei* was significantly (*p* < 0.05) upregulated, which indicated that recombinant *L. casei* could activate the innate immune system to trigger the cell immune response and inflammatory response. Furthermore, recombinant *L. casei* was able to survive the intestinal environment and colonize in intestine mucosal. The study showed that after being challenged by *A. veronii*, fish administered with Lc-pPG-1-FlaA (70%) and Lc-pPG-2-FlaA (50%) had higher survival rates compared to Lc-pPG and PBS, indicating that recombinant *L. casei* might prevent *A. veronii* infection by activating the immune system to trigger immune responses. We demonstrated that flagellin as an antigen of vaccine, is acceptable for preventing *A. veronii* infection in fish. The recombinant *L. casei* expressing FlaA may be a novel mucosal vaccine for treating and controlling *A. veronii*.

## 1. Introduction

*Aeromonas veronii*, a gram-negative opportunistic pathogen, is associated with infections in both fish and mammals [1,2]. *A. veronii* is widely distributed in fresh water, sludge, sewage, soil and other natural environments [3]. The main aquatic animals infected by *A. veronii* include goldfish [4], Nile tilapia (*Oreochromis niloticus*) [5], Pacific red snapper (*Lutjanus peru*) [6] and turtles (*Trionyx sinens*) [7], resulting in serious economic losses in aquaculture and creating a potential threat to aquatic food safety [8,9,10,11]. By using antibiotics, it is possible to treat diseases induced by *Aeromonas*. However, antibiotics may harm water quality and human health, thus it is necessary to develop a novel vaccine for preventing *A. veronii* infection [12].

Mucosal tissues are important for disease prevention and cure. There are several reports that suggest that probiotics have positive effects in the prevention of pathogen infection and improvement of the mucosal immune response [13,14,15]. *Lactobacillus* are “generally regarded as safe (GRAS)” microorganisms, and are used for antigen expression owing to their ability to present compounds on the mucous membrane and colonize in the gut [16]. Bacterial flagella are complex nano-machines motility that allows them to move towards nutrients and more favorable environments. Furthermore, flagellin is an important antigen and it is the target of the innate immune system through toll-like receptor 5 (TLR5) [17]. Therefore, flagellin could be considered as a candidate for a novel vaccine.

In this study, *Lactobacillus casei* CC16 strains expressing *A. veronii* antigen flagellin A (FlaA) were successfully constructed. To evaluate the effects of orally administered recombinant *L. casei*, we investigated the immune response induced by recombinant *L. casei* and its protective effects after being challenged with *A. veronii*. The results showed that recombinant *L. casei* was able to activate the innate immune system to induce an immune response and provide protection from *A. veronii* infection.

## 2. Results

### 2.1. Construction of the Recombinant L. casei

**Figure 1:**
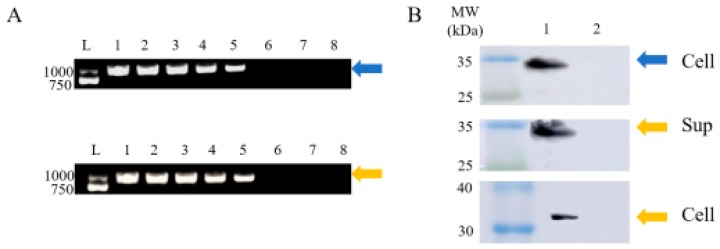
Expression of flagellin A (FlaA) by recombinant identified by PCR and Western blotting assay. (**A**) PCR result of recombinant *L. casei* expressing FlaA. L: DNA ladder(bp), Lane 1–5: PCR product of Cm-resistant clones, Lane 6: PCR product of Lc-pPG, Lane 7: PCR product of *L. casei*, Lane 8: negative control. (**B**) Western blotting analysis. The orange and blue arrow are Lc-pPG-1-FlaA and Lc-pPG-2-FlaA, respectively. Cellular extracts (Cell) and culture supernatants (Sup) were analyzed with western blotting. MW indicated molecular mass markers (kDa). The immunoreactive bands indicated that the recombinant *L. casei* secreted FlaA (33kDa) in cell lysates and supernatants, recombinant *L. casei* (Lane: 1) and Lc-pPG (Lane: 2).

The recombinants of *L. casei* were obtained by screening Cm-resistant clones and confirmed by PCR and sequence analysis. The sequence was precisely the same as the designed sequence. The PCR results (Figure 1A) and sequence analysis illustrated that the recombinant plasmids, Lc-pPG-1-FlaA and Lc-pPG-2-Fla, had been successfully constructed in *L. casei*.

### 2.2. Western Blotting

Cell protein extracts from Lc-pPG-1-FlaA, Lc-pPG-2-FlaA and Lc-pPG were analyzed by Western blotting. Results indicated that the modified FlaA was successfully expressed. There was a band with 31kDa detected in the cell lysates of Lc-pPG-1-FlaA, the immunoreactive bands were detected in both of the cell lysates and supernatant of Lc-pPG-2-FlaA, whereas no band was observed in the Lc-pPG (Figure 1B).

### 2.3. Surface Expression of the Modified FlaA

**Figure 2:**
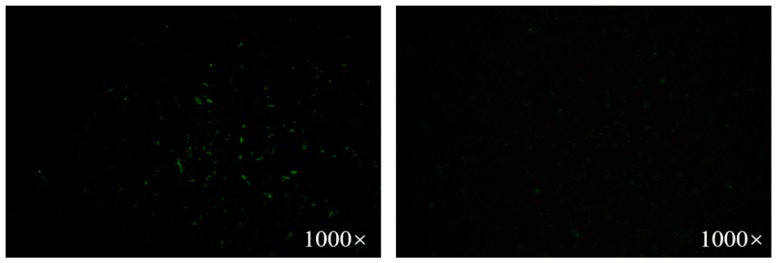
Immunofluorescence assay. Lc-pPG-1-FlaA (left) and Lc-pPG (right), magnification: ×1000. There was significant green fluorescence on the cell surface of Lc-pPG-1-FlaA and no immunofluorescence reaction on the Lc-pPG cell surface.

Immunofluorescence microscopy was used to confirm the expression of FlaA protein on the surface of recombinant Lc-pPG-1-FlaA. Briefly, Lc-pPG-1-FlaA and Lc-pPG were treated as described. As shown in Figure 2, the FlaA proteins were visible on the surface of Lc-pPG-1-FlaA. These indicated that Lc-pPG-1-FlaA produced FlaA proteins and these were displayed on the surface of bacterial cells.

### 2.4. Humoral Immune Parameters

**Figure 3:**
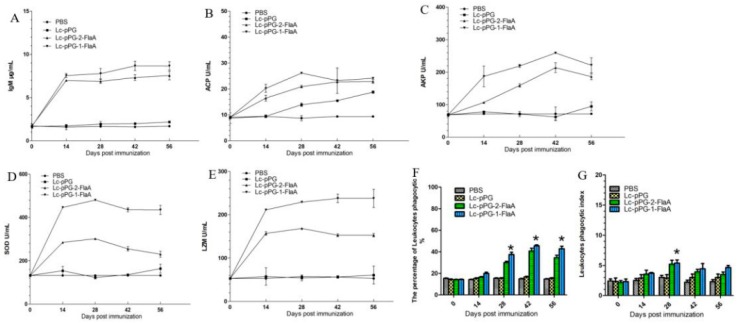
Humoral immune response stimulated by recombinant *L. casei*. Changes in serum IgM (**A**): acid phosphatase (ACP) (**B**): alkaline phosphatase (AKP) (**C**); superoxide dismutase (SOD) (**D**); and lysozyme (LZM) activity (**E**) in peripheral blood of common carp (*n* = 3 fish/group) after oral administration. Leukocyte phagocytic percentage (PP) (**F**) and index (PI) (**G**) of common carp (*n* = 3 fish/group) after immunization. Data are presented as mean ± SD compared to PBS control. *: *p* < 0.05.

**Figure 4:**
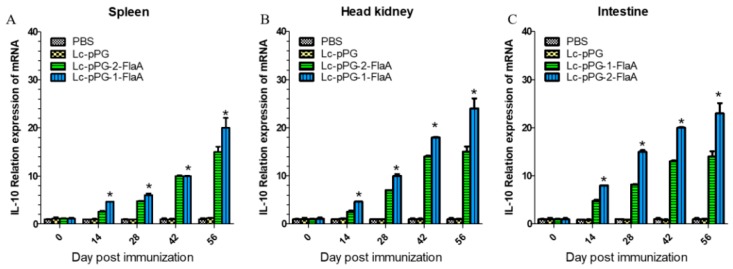
The mRNA expression levels of IL-10 in the spleen (**A**), head kidney (**B**), and intestine (**C**) were analyzed by qRT-PCR (*n* = 3 fish/group). Data presented as mean ± SD compared to PBS control. *: *p* < 0.05.

The effects of recombinant *L. casei* on the IgM of serum are shown in Figure 3A. Common carp orally administrated with Lc-pPG-1-FlaA and Lc-pPG-2-FlaA showed significantly higher IgM levels as compared to the control (*p* < 0.05) on day 28. Similarly, higher serum ACP, AKP, SOD and LZM activity were observed in fish administrated with Lc-pPG-1-FlaA and Lc-pPG-2-FlaA on day 56 compared to the control (*p* < 0.05) (Figure 3B–E). There was no significant difference between PBS and the Lc-pPG group. The phagocytic activity and index were significantly higher (*p* < 0.05) in fish administrated with Lc-pPG-1-FlaA and Lc-pPG-2-FlaA, compared to PBS (Figure 4F–G).

### 2.5. Expression of Immune-Related Genes

**Figure 5:**
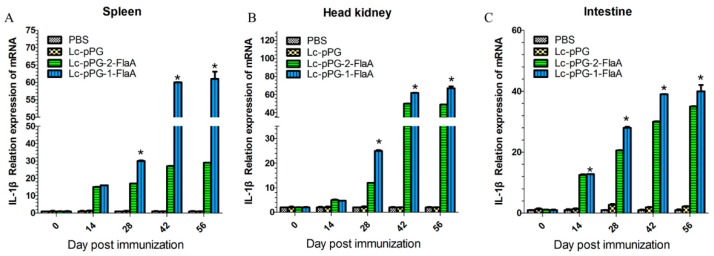
The mRNA expression levels of IL-1β in the spleen (**A**), head kidney (**B**) and intestine (**C**) were analyzed by qRT-PCR (n = 3 fish/group). Data presented as mean ± SD compared to PBS control. *: *p* < 0.05.

**Figure 6:**
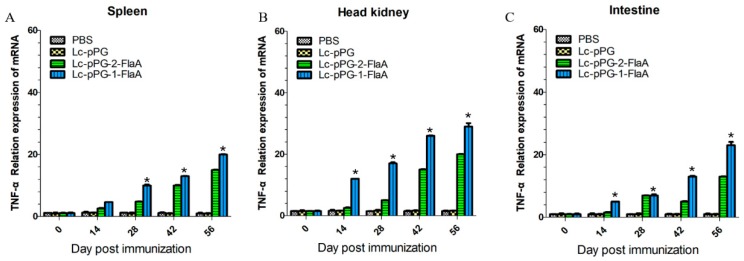
The mRNA expression levels of TNF-α in the spleen (**A**), head kidney (**B**) and intestine (**C**) were analyzed by qRT-PCR (*n* = 3 fish/group). Data presented as mean ± SD compared to PBS control. *: *p* < 0.05.

**Figure 7:**
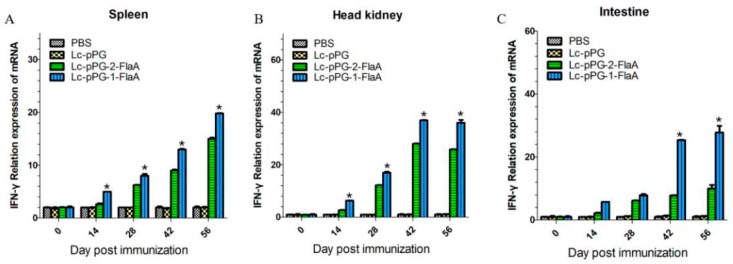
The mRNA expression levels of IFN-γ in the spleen (**A**), head kidney (**B**) and intestine (**C**) were analyzed by qRT-PCR (*n* = 3 fish/group). Data presented as mean ± SD compared to PBS control. *: *p* < 0.05.

**Figure 8:**
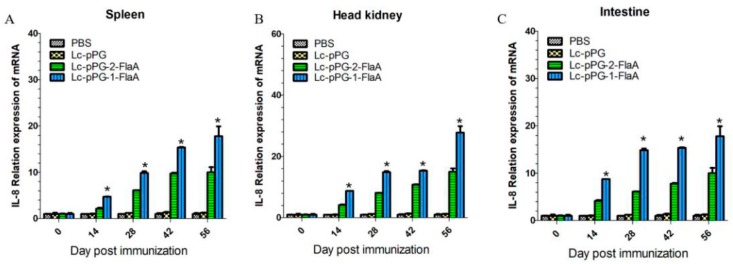
The mRNA expression levels of IL-8 in the spleen (**A**), head kidney (**B**) and intestine (**C**) were analyzed by qRT-PCR (*n* = 3 fish/group). Data presented as mean ± SD compared to PBS control. *: *p* < 0.05.

To further examine the effect of recombinant *L. casei* in the stimulation of immune responses, immune-related genes IFN-γ, IL-10, IL-1β, TNF-α and IL-8 were examined by qRT-PCR analysis. IL-10 expression was significantly increased in the head kidney (HK), spleen and intestine in the Lc-pPG-1-FlaA and Lc-pPG-2-FlaA groups on day 14 compared with the other groups (*p* < 0.05, Figure 4). As shown in Figure 5, a significant increase in IL-1β expression in the intestine in the Lc-pPG-2-FlaA and Lc-pPG-1-FlaA groups was observed on day 56, and there were no significant differences in the spleen and HK on day 14 in the Lc-pPG-1-FlaA and Lc-pPG-2-FlaA groups (*p* > 0.05). However, on day 42, a significant upregulation in the HK, spleen and intestine was observed in the Lc-pPG-1-FlaA and Lc-pPG-2-FlaA groups (*p* < 0.05). Also, rapid changes in TNF-α expression were observed in the Lc-pPG-2-FlaA and Lc-pPG-1-FlaA groups (Figure 6). TNF-α expression of recombinant *L. casei* in the intestine was significantly increased compared with other groups (*p* < 0.05, Figure 6C). Almost no significant difference was observed in IFN-γ expression in the spleen and HK at day 28 (*p* > 0.05, Figure 7), while on day 56, IFN-γ expression in the spleen, HK and intestine reached their peak, and there was a two-fold upregulation in the HK and intestine on day 56. Higher expression of IL-8 in the HK and intestine was observed on day 42 (*p* < 0.05, Figure 8).

### 2.6. Colonization of Recombinant L. casei in the Fish Intestine

**Figure 9:**
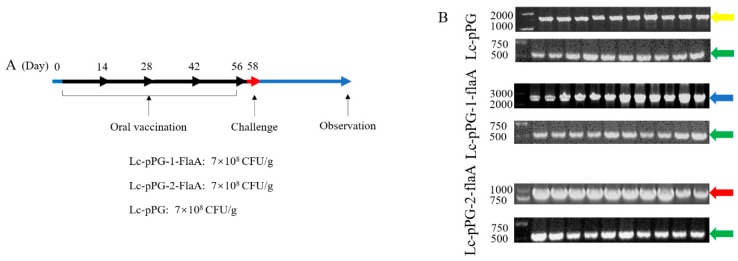
Colonization of recombinant *L. casei* expressing FlaA in the intestine of common carp. (**A**) Experimental schedule. (**B**) Ten single colonies were randomly picked from each plate for PCR with the pPG-vector primer and *dnaA* primer. Bands indicated by red (900 bp), blue (2400 bp), yellow (1600 bp) and green arrows (515 bp) were consistent with putative sequences analyzed by DNA sequencing. L: DNA ladder (bp), Lane 1–10: PCR product of recombinant *L. casei*.

To detect colonization of recombinant *L. casei* in the intestine, the entire intestine was homogenized and the growth of the colonies was observed on MRS supplemented with Cm. More colony-forming units of Lc-pPG-1-FlaA were observed as compared with Lc-pPG-2-FlaA (Figure 9A) whereas no colonies were observed on plates containing homogenates of PBS and Lc-pPG. To identify the colonies of recombinant *L. casei*, two primer pairs were used for PCR-colony screening. PCR results showed that all randomly selected colonies’ PCR products (515bp) were amplified with the *dnaA* primer pair. PCR products of Lc-pPG (1600bp), Lc-pPG-1-FlaA (2400bp) and Lc-pPG-2-FlaA (900bp) were amplified with the pPG-Vector primer pair (Figure 9B).

### 2.7. Challenge Test

**Figure 10:**
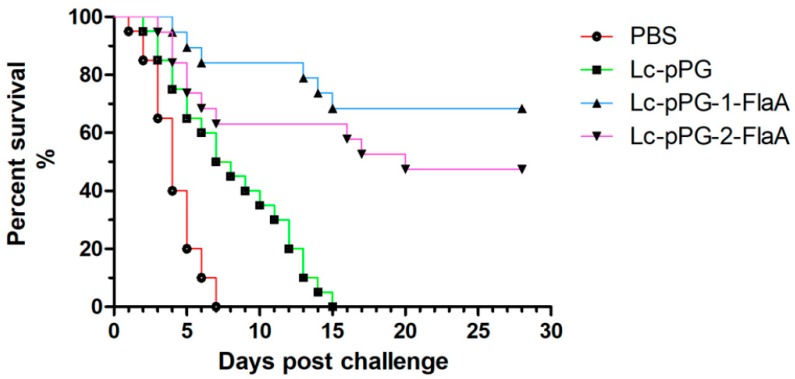
Survival rate of common carp challenged by *A. veronii* TH0426 after oral immunization with Lc-pPG-1-FlaA, Lc-pPG-2-FlaA, Lc-pPG and PBS at day 58. Thirty fish/group were used to record the survival rate for 28 days.

To evaluate protective immunity of recombinant *L. casei*, a challenge test was performed. At the end of the monitoring period, the survival rate of the common carp immunized with Lc-pPG-1-FlaA, Lc-pPG-2-FlaA, Lc-pPG and PBS groups was 70%, 50%, 0% and 0%, respectively (Figure 10).

## 3. Discussion

*A. veronii* is a serious pathogen that causes high mortality rates and economic losses in the aquaculture industry [18]. Although antimicrobials can treat bacterial disease, their widespread and excessive use exacerbates the development of drugs that are resistant to bacteria [19]. This is harmful for the sustainable development of aquaculture.

The development of vaccines is of practical significance for aquaculture. However, while vaccination by injection has been recognized as a efficient method to induce protective immunity [20,21,22], injection is not suitable for vaccinating farmed fish due to handling stress, high labor costs and the number of fish [23]. In teleosts, the gut is one of the main mucosal surfaces and immune barriers [24]. Therefore, oral administration induces protective immunity more effectively through mucosa-associated tissues [23]. In this study, for the first time we engineered and evaluated the recombinant *L. casei* expressing FlaA protein of *A. veronii* to induce an immunity response and immunogenicity in common carp.

Research on *Aeromonas* flagella shows that the helical filament of *Aeromonas* polar flagellum comprises two flagellin subunits known as FlaA and FlaB [25,26,27]. Flagellin is a target of that activates the innate immune system through toll-like receptor 5 (TLR5) [28,29]. After TLR5 recognizes flagellin, the nuclear factor (NF)-κB is activated to produce and secrete pro-inflammatory cytokines and chemokines for an effective immunity response [30]. In this study, we compared the immunogenicity of two recombinants of *L. casei* expressing FlaA after fish were inoculated by oral administration. The ELISA results showed that serum IgM was significantly increased after oral immunization on day 28 in the Lc-pPG-1-FlaA group, and significant enhancement of lysozyme, SOD and AKP in serum was also observed in this group on day 56. Similar results were observed in the Lc-pPG-2-FlaA group, although the levels of the humoral immune indexes were lower than in the Lc-pPG-1-FlaA group. The phagocytic activity and index were significantly higher in the Lc-pPG-1-FlaA group. We hypothesized that the FlaA protein expressed by recombinant *L. casei* could reach the intestine mucosal tissues and activate the innate immune system. Moreover, the FlaA protein expressed by Lc-pPG-2-FlaA may be partly accessible to the intestine mucosal surface and effectively activate the innate immune system, which could result in the higher level of immune indexes compared to that induced by Lc-pPG-2-FlaA. In previous studies, aquatic animals fed with probiotics showed significant enhancement of ACP, SOD, AKP and LZM activity [31,32,33,34]. These results indicated that recombinant *L. casei* can activate the innate immune system through oral administration, as we found in our study.

Cytokines play a key role in the host’s defense mechanisms and innate immune system. IL-1β, TNF-α is an important component of early inflammatory events [35]. IFN-γ is one of most important cytokines to activate macrophages in order to inhibit replication of parasites [36]. Our results showed strong expression of IL-1β, TNF-α and IFN-γ in the spleen, head kidney and intestine in the recombinant *L. casei* groups, which is consistent with studies of cytokines upregulation induced by recombinant flagellins [30]. IL-8 is a chemokine induced through NF-κB [37]. IL-10 is a potent anti-inflammatory cytokine produced by monocytes, natural killer (NK) cells, macrophages and dendritic cells [38,39]. The recombinant *L. casei* groups showed stronger expression of IL-10 and IL-8 in the spleen, HK and intestine of fish compared with the PBS group. A similar phenomenon was observed in the enhancement of IL-10 expression in fish treated with probiotics [32,40]. Higher IL-8 expression was observed in a previous study [40]. The results indicated that recombinant *L. casei* could effectively activate the immune system to secrete cytokine and chemokine for an effective immune response.

Intestinal microbiota play a key role in the intestinal barrier. The intestinal microbial composition is partially impacted by food and the environment. Colonization of recombinant *L. casei* in fish using oral administration was evaluated. The colonies’ PCR results showed that colonies from recombinant *L. casei* could colonize the intestine. In previous studies, *L. lactis* was able to survive the intestinal environment [41,42]. This study indicated that recombinant *L. casei* could release antigens and survive the intestinal environment, and that recombinant *L. casei* is acceptable as a novel therapeutic strategy to enhance host immunity against pathogens.

The results of the survival study showed that recombinant *L. casei* provided strong protection for common carp against the challenge of *A. veronii*; the survival rate of fish administered with Lc-pPG-1-FlaA (70%) was higher than those administered with Lc-pPG-2-FlaA (50%). We hypothesized that recombinant *L. casei* could activate the immune system through oral administration, survive the intestinal environment and defend against pathogens as an intestinal barrier. In further research, the intestinal microbiota composition and the immune mechanisms of fish affected by recombinant *L. casei* need to be explored. To develop a novel vaccine it is necessary to explore the detailed mechanisms of how *lactobacillus* activates the immune system.

## 4. Materials and Methods

### 4.1. Fish and Ethics Statement

All experimental protocols for this research were approved(15 September, 2017) by the Regulations for Animal Experimentation of Jilin Agricultural University (JLAU08201409). Specific pathogen-free and clinically healthy common carp (mean weight 55 ± 3 g) were obtained from fisheries (Jilin, China). The fish were acclimated to the experimental environment for two weeks, according to Safari et al. [43]. Fish were fed with commercial diet twice a day at a feeding rate of 1% body weight. The water temperature was 25 ± 2 °C, and the water pH was at 7.0 ± 0.5.

### 4.2. Bacterial Strains, Plasmids and Growth Conditions

*Lactobacillus casei* CC16 was isolated from the intestine of common carp, and grown in De Man, Rogosa and Sharpe (MRS) medium (Thermo Fisher Scientific, Oxoid, UK) at 30 °C without shaking. The *Escherichia coli*-*Lactobacillus* shuttle vector pPG-1 and pPG-2 have been described in our previous study [44]. The competent cells, Escherichia coli MC1061, were grown in Luria-Bertani (LB) medium for cloning of the plasmids at 37 °C with shaking. Chloramphenicol (Cm) was utilized at a final concentration 10 μg/mL when it was necessary. *Aeromonas veronii* TH0426 strain was isolated from the farmed yellow catfish *Pelteobagrus fulvidraco* as described by Kang et al. [45].

### 4.3. Construction of Recombinant L. casei Expressing FlaA Gene

**Figure 11:**
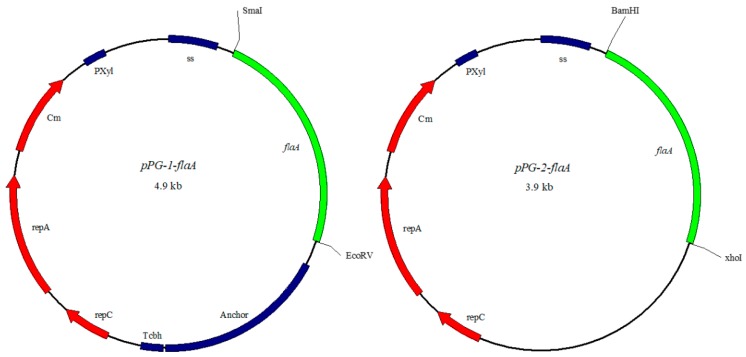
Expression of FlaA protein on *L. casei*. Plasmid maps of the surface-displayed expressing, pPG-1-FlaA(left), and secretion expressing plasmid pPG-2-FlaA(right).

The FlaA (903bp) gene was amplified from *Aeromonas veronii* TH0426 (CP12504.1) by PCR using the primer pairs shown in Table 1 and gel purified. Each processed fragment was ligated to pPG-1 and pPG-2 cut with *Sma* I/*BamH* I and *EcoR* V/*Xho* I, respectively (Figure 11). The recombinant plasmids (pPG-1-FlaA and pPG-2-FlaA) and pPG were transformed into *L. casei* CC16 by electroporation, according to Hou et al. [46]. The *L. casei* CC16 containing pPG without FlaA gene was used as vector control.

### 4.4. Western Blotting Assay

For the expression analysis of the modified FlaA, the recombinant Lc-pPG-1-FlaA, Lc-pPG-2-FlaA and Lc-pPG were grown in MRS medium supplemented with 10 μg/mL of Cm. Xylose was added to the culture medium to a final concentration of 10 g/L to induce antigen expression. After induction at 30 °C for 10 h, bacterial cells or supernate (a 10-fold concentration) were examined by SDS-PAGE and transferred to nitrocellulose membrane as in our previous study [44].

### 4.5. Expression of FlaA on the Cell Surface

Immunofluorescence was used to determine the expression of FlaA protein on the surface of Lc-pPG-1-FlaA as in previous studies [45,46]. Briefly, Lc-pPG-1-FlaA, Lc-pPG-2-FlaA and Lc-pPG were grown in MRS broth as described, the Lc-pPG was used as negative. Cultures of 500 μL were collected by centrifugation at 5000 rpm for 5 min, and the pellets were washed twice and resuspended in 50 μL PBS containing 1% bovine serum albumin (BSA, Gibco, Thermo Fisher Scientific, UK) and mouse anti-FlaA antiserum (1:200 dilution, antiserum was prepared and saved by laboratory member) at 37 °C for 1 h. The cells were incubated with fluorescein isothiocyanate (FITC, CWBIO, Beijing, China)-conjugated goat anti-mouse IgG at 37 °C for 2 h.

### 4.6. Oral Immunization and Sample Collection

The recombinant *L. casei* was grown in MRS supplemented with Cm and xylose as previous described. Overnight cultures were mixed with commercial basal diet feed. After the diet feed were oven-dried at 40 °C for 6 h, the diet feed containing 10^9^ colony forming unit (CFU)/g were kept at 4 °C prior to feeding. A total of 200 common carp were randomly distributed into four groups, resulting in 50 fish in each cage. The fish were fed with diet feed containing Lc-pPG-1-FlaA, Lc-pPG-2-FlaA, Lc-pPG and PBS, respectively. The feeding trial was conducted for 8 weeks, and the immune protocol administration is shown in Figure 10. Three fish in each group were anaesthetized with MS-222 (Sigma, Darmstadt, Germany) on day 0, 14, 28, 42 and 56 to draw blood from caudal veins. Furthermore, pieces of the spleen, head kidney (HK) and intestine were rapidly excised, frozen in liquid nitrogen, and stored at −80 °C until RNA extraction.

### 4.7. Phagocytic Activity of Leukocyte

Leukocyte phagocytic function was slightly modified as follows: after blood collection, 200 μL of blood from each fish group was dropped into heparinized centrifuge tubes, to which 100 μL bacterial suspension of *Staphylococcus aureus* was added before shaking, according to Avtalion et al. [47]. The tubes were kept at 28 °C in a water bath for 30 min, and shaken every 10 min in order to centrifuge as recommended by Cai et al. [48]. The supernatant was discarded, and the upper layer of the precipitate was used to make blood slides. Slides were air dried, fixed in methanol and stained with Giemsa solution (Solarbio, Beijing, China). Slides were viewed under oil immersion at 100×. Approximately 100 cells were counted in random fields of view and the phagocytic percentage (PP) and phagocytic index (PI) were determined as Li described [49], as follows:

PP (%) = (number of cells involved in phagocytosis / total number of cells) × 100(1)

PI = number of phagocytosed bacteria / number of cells involved in phagocytosis(2)

### 4.8. Enzyme-Linked Immunosorbent Assay (ELISA)

Serum IgM, lysozyme (LZM) activity, acid phosphatase (ACP), superoxide dismutase (SOD) and alkaline phosphatase (AKP) were evaluated using ELISA kits (Nanjing Jiancheng Bioengineering Institute, China).

### 4.9. Real-Time PCR Analysis

Simply *p* total RNA kit (Bioflux-Bioer, Hangzhou, China) was used for total RNA extraction from different tissues. The concentration of RNA samples was examined using Nanodrop 2000c (Thermo Fisher Scientific, Foster city, CA, USA), and the complementary DNA (cDNA) was synthesized using PrimeScript™ RT reagent kit with gDNA eraser (Takara, Dalian, China). The real-time PCR was performed with Applied Biosystems^®^ 7500 Real-Time PCR systems (Thermo Fisher Scientific, Foster city, CA, USA), using SYBR Green Master Mix (Takara, Dalian, China). The primers of the immune-related genes studied and the β-actin are shown in Table 1.

### 4.10. Colonization of Recombinant L. casei in the Fish Intestine

To detect the colonization of recombinant *L. casei* in the fish intestine, the intestine of common carp that were fed for 14 d and then starved for 7 d was collected. The samples were homogenized in sterile PBS and serial-diluted (100 μL of 10^4^-fold dilutions) for plating on MRS Cm agar plate. The homogenates were incubated anaerobically at 30 °C for 24 h, and 10 signal colonies were randomly selected and used for PCR. The primers were as follow: pPG-vector primer pairs (forward: 5′ TGCTTCTGCTGTATCTACTGTTAGC 3′; reverse: 5′ TCTCATTGAGAAGATTGCCGAAA 3′); *L. casei* housekeeping gene *dnaA* primer (forward: 5′ TCTGTTTATTTATGGTGGCG 3′; reverse: 5′ CTGCGGTCATCAAGTTTCA 3′). PCR products were verified by DNA sequencing analysis.

### 4.11. Challenge Test

All the vaccinated fish were injected intraperitoneally with 200 μL of 5×10^6^CFU (5 LD_50_ dose) of *A. veronii* TH0426 strain on day 58 (2 days after the final oral immunization). Fish injected with 200 μL PBS were used as the negative control group. The fish challenged with *A. veronii* were monitored for 28 days and the survival rate was analyzed post challenge in all the groups.

### 4.12. Data Analysis

Results were presented as means ± standard deviation (SD). Statistical analysis was performed using SPSS v16.0 software and GraphPad PRISMM v5.0. All data were subjected to a one-way analysis of variance. In all cases, significant differences were considered as *p* < 0.05.

## 5. Conclusions

In conclusion, our study demonstrated that humoral immune parameters, ACP, AKP, SOD, IgM and LYZ were significantly increased in fish immunized with recombinant *L. casei*. The expression of IL-10, IL-8, IL-1β, TNF-α and IFN-γ in fish tissue was regulated by oral administration of recombinant *L. casei*. Recombinant *L. casei* was able to survive the intestinal environment and release antigen, activate immune systems, and provide protection for common carp against *A. veronii*. Our study indicates that recombinant *L. casei* may be acceptable as a novel vaccine against *A. veronii*.

## Figures and Tables

**Table 1:** Primers used in this study.

Primer	Sequence(5’-3’)	Accession	PCR Product (bp)
IL-8	F: CATTCAGAGCCAGCAATT	AB470924.1	135
	R: CACCCAGTCTTTAGTAGGAT		
IL-10	F: AACTGATGACCCGAATGGAAAC	JX524550.1	143
	R: CACCTTCTCCCAGTCGTCAAA		
IL-1β	F: CATCAAAGAAATCGCTCCTG	AB010701	133
	R: GCAAGGTCTGCCTGGTCT		
TNF-α	F: TTATGTCGGTGCGGCCTTC	AJ311800.2	101
	R: AGGTCTTTCCGTTGTCGCTTT		
IFN-γ	F: AACAGTCGGGTGTCGCAAG	AB376666.1	141
	R: TCAGCAAACATACTCCCCAG		
β-actin	F: CAAGATGATGGTGTGCCAAGTG	M24113.1	352
	R: TCTGTCTCCGGCACGAAGTA		
pPG-1-FlaA	F:AACCCGGGATGGGCCTTTTTATCAACACTAACG	CP012504.1	903
	R:CCGATATCTTAGCCTTGCAGCAGCTGAAGTGCT		
pPG-2-FlaA	F: CGGGATCCATGGGCCTTTTTATCAACACTAACG	CP012504.1	903
	R:CCCTCGAGTTAGCCTTGCAGCAGCTGAAGTGCT		
